# Average symptom trajectories following incident radiographic knee osteoarthritis: data from the Osteoarthritis Initiative

**DOI:** 10.1136/rmdopen-2016-000281

**Published:** 2016-07-04

**Authors:** Rebecca Whittle, Kelvin P Jordan, Elaine Thomas, George Peat

**Affiliations:** Arthritis Research UK Primary Care Centre, Research Institute for Primary Care & Health Sciences, Keele University, Staffordshire, UK

**Keywords:** Knee Osteoarthritis, Osteoarthritis, Epidemiology

## Abstract

**Introduction:**

Previous research has identified the existence of a prodromal phase of symptom worsening beginning on average 2–3 years prior to the first appearance of radiographic knee osteoarthritis (OA). The current study extends these observations to investigate the trajectory of self-reported pain, stiffness, function and other symptoms following the incidence of radiographic OA.

**Methods:**

Data were from the incidence cohort of the Osteoarthritis Initiative public use data sets. Cases were defined as knees without symptoms at enrolment, which developed incident radiographic OA (Kellgren and Lawrence grade ≥2) at any of the first 4 annual follow-up visits. Symptoms investigated were knee-specific Western Ontario & McMaster Universities Osteoarthritis Index and Knee injury and Osteoarthritis Outcome Score subscale scores and individual items, available up to 3 years before and 5 years after the incidence of radiographic OA. Trajectories of having at least one of the symptoms from a subscale, and for each individual symptom over time, were fitted using multilevel logistic regression models.

**Results:**

The probability of symptoms following the initial prodromal phase generally stabilised, whereas the probability of moderate, severe or extreme symptoms was consistently low. Two exceptions were pain frequency, which increased greatly in the lead up to incidence, then decreased slightly, and audible joint sounds, which had a much higher overall probability, and after increasing prior to incident radiographic OA, stabilised then started to increase again at 5 years.

**Conclusions:**

Following an increase in the risk of symptoms during the prodromal phase, this risk does not continue to increase in the period up to 5 years after the incidence of radiographic OA.

**Key messages**

**What is already known about this subject?**

- In adults at high risk of knee osteoarthritis (OA) symptoms begin to appear or worsen, on average, 2-3 years prior to the first appearance of incident radiographic knee OA.

**What does this study add?**

- We extended these findings forward in time and demonstrated that following an increase in the risk of symptoms prior to the incidence of radiographic knee OA, this risk does not continue to increase in the period up to 5 years after incidence.

**How might this impact on clinical practice?**

- Patients undergoing an underlying transition in OA disease state may experience episodic symptom worsening but this does not inevitably presage further symptom decline in the short-to-medium term.

## Introduction

Observations by Felson *et al*^1^ support the hypothesis that disease progression in knee osteoarthritis (OA) follows a pattern of inertia in which knees showing recent incident change in X-ray are at higher risk of further X-ray progression. Given well-documented structure–pain discordance in OA, it is less clear whether patients' reported experience of pain and function follow a similar pattern. This is important since the experience of symptoms and functional limitation drive help-seeking and should therefore feature in preventive strategies based on early diagnosis.

In a previous analysis of repeated-measures data in a cohort of adults at high risk of knee OA, we found that symptoms began to appear or worsen, on average, 2–3 years prior to the first appearance of incident radiographic knee OA (ROA: defined as Kellgren and Lawrence (KL) grade ≥2)—a ‘prodromal phase’.^2^ Here, we extend these observations forward in time to establish if this increase in symptoms during the prodromal phase is followed by further symptom worsening.

## Methods

### Data set

We used data from the Osteoarthritis Initiative (OAI)^3^ (available for public access at http://www.oai.ucsf.edu/). Between 2004 and 2006, 3284 participants aged 45–79 years were enrolled in the ‘incidence subcohort’ of the OAI if they were at high risk of developing symptomatic ROA. Individuals with rheumatoid arthritis or other inflammatory arthritis were excluded. Measures recorded from self-complete questionnaires, personal interviews, physical examinations and plain radiography were collected on enrolment and were repeated at annual clinic visits. All participants signed informed consent, and the study was approved by the institutional review board.

### Selection of participants

Cases were defined as knees without symptoms (defined as knee pain, aching or stiffness: more than half the days of a month, past 12 months) on enrolment into the OAI, which had developed incident ROA (KL grade ≥2), defined as the new onset of combined definite osteophyte and joint space narrowing in the tibiofemoral joint^4^ (ascertained from fixed-flexion knee radiographs), at any of the first four annual follow-up visits. Cases were assigned a common baseline time point, t0, corresponding to when incident ROA was first identified. Knees that were surgically replaced were censored at the last visit before the knee replacement was recorded.

### Outcome measures

Outcomes, measured annually up to 3 years before and 5 years after incident ROA, were the Western Ontario & McMaster Universities Osteoarthritis Index (WOMAC^5^), Pain, Physical Function and Stiffness subscales and Knee injury and Osteoarthritis Outcome Score (KOOS^6^), Pain and Other Symptoms subscales (dichotomised into at least one item in the subscale rated ‘moderate’, ‘severe’ or ‘extreme’ vs all items in the subscale rated ‘none’ or ‘mild’) and 34 individual items from the WOMAC and KOOS scales (dichotomised: ‘none’/‘mild’ vs ‘moderate’/‘severe’/‘extreme’; see ref. 2). For the current analyses, we used the knee-specific subscale scores and individual items as previously studied in the prodromal phase.^2^

### Statistical analysis

Trajectories of the probabilities of symptoms over time were estimated with random intercept multilevel logistic regression models,^7^ adjusting for age and gender, treating each knee as an individual case while accounting for correlation between knees within people. Complete case analysis was performed.

## Results

One hundred and sixty-nine cases of incident ROA were recorded in 161 participants during the first 4 years of follow-up of the OAI cohort (68, 31, 47 and 23 knees at years 1, 2, 3 and 4, respectively). At cohort entry: mean age 65.2 years (SD 9.2); 69% female; mean BMI 29.2 kg/m^2^ (SD 4.6); 42.6% previous knee injury; 20.7% previous knee surgery; 72% with KL=1. Approximately 25% of knees had at least one symptom (individual item) present at t0 on each WOMAC subscales, reducing to ∼15% by t0+1 for Pain and Stiffness and 23% for Physical Functioning with little further change by t0+5 (table 1).

**Table 1:** Number (%) of knees with symptoms at each time point and ORs (95% CIs) for association of symptoms with time from incident radiographic OA from multilevel models fitted to subscale scores, data from the Osteoarthritis Initiative, USA, 2004–2012

	WOMAC Physical Function		WOMAC Pain		WOMAC Stiffness		KOOS Pain		KOOS Other Symptoms	
Time	N (%)	OR (95% CI)	N (%)	OR (95% CI)	N (%)	OR (95% CI)	N (%)	OR (95% CI)	N (%)	OR (95% CI)
t0–3	12 (17.1)	0.45 (0.19 to 1.07)	8 (11.4)	0.35 (0.13 to 0.93)	9 (12.9)	0.46 (0.21 to 1.01)	17 (26.2)	0.27 (0.13 to 0.56)	13 (18.6)	0.18 (0.08 to 0.41)
t0–2	14 (14.3)	0.37 (0.17 to 0.81)	11 (11.2)	0.38 (0.16 to 0.88)	9 (9.2)	0.33 (0.15 to 0.70)	20 (22.2)	0.22 (0.11 to 0.43)	25 (25.5)	0.39 (0.20 to 0.75)
t0–1	44 (26.8)	0.96 (0.54 to 1.69)	35 (21.3)	0.97 (0.53 to 1.76)	30 (18.4)	0.70 (0.41 to 1.19)	62 (39.7)	0.61 (0.37 to 1.02)	51 (32.7)	0.59 (0.34 to 1.01)
t0	46 (27.2)	Ref.	37 (22.0)	Ref.	40 (24.0)	Ref.	79 (48.2)	Ref.	66 (39.3)	Ref.
t0+1	36 (22.6)	0.74 (0.41 to 1.34)	26 (16.4)	0.63 (0.33 to 1.19)	23 (14.6)	0.55 (0.31 to 0.97)	64 (42.7)	0.75 (0.45 to 1.24)	54 (34.6)	0.81 (0.47 to 1.39)
t0+2	36 (23.5)	0.82 (0.45 to 1.49)	28 (18.3)	0.75 (0.40 to 1.41)	23 (15.0)	0.59 (0.33 to 1.05)	54 (37.8)	0.56 (0.33 to 0.94)	59 (38.8)	1.06 (0.62 to 1.80)
t0+3	32 (24.2)	0.87 (0.47 to 1.64)	16 (12.1)	0.42 (0.20 to 0.89)	26 (19.7)	0.83 (0.47 to 1.47)	48 (38.1)	0.58 (0.33 to 1.00)	47 (35.6)	0.94 (0.53 to 1.67)
t0+4	21 (25.3)	0.95 (0.46 to 1.98)	14 (16.9)	0.74 (0.33 to 1.65)	14 (16.9)	0.68 (0.34 to 1.38)	30 (38.5)	0.60 (0.31 to 1.15)	23 (28.1)	0.63 (0.31 to 1.27)
t0+5	12 (21.1)	0.64 (0.26 to 1.57)	9 (15.8)	0.63 (0.24 to 1.62)	8 (14.0)	0.54 (0.23 to 1.31)	21 (38.9)	0.60 (0.28 to 1.27)	24 (42.9)	1.57 (0.72 to 3.43)

#KOOS, Knee injury and Osteoarthritis Outcome Score; OA, osteoarthritis; WOMAC, Western Ontario &McMaster Universities Osteoarthritis Index.

**Figure 1:**
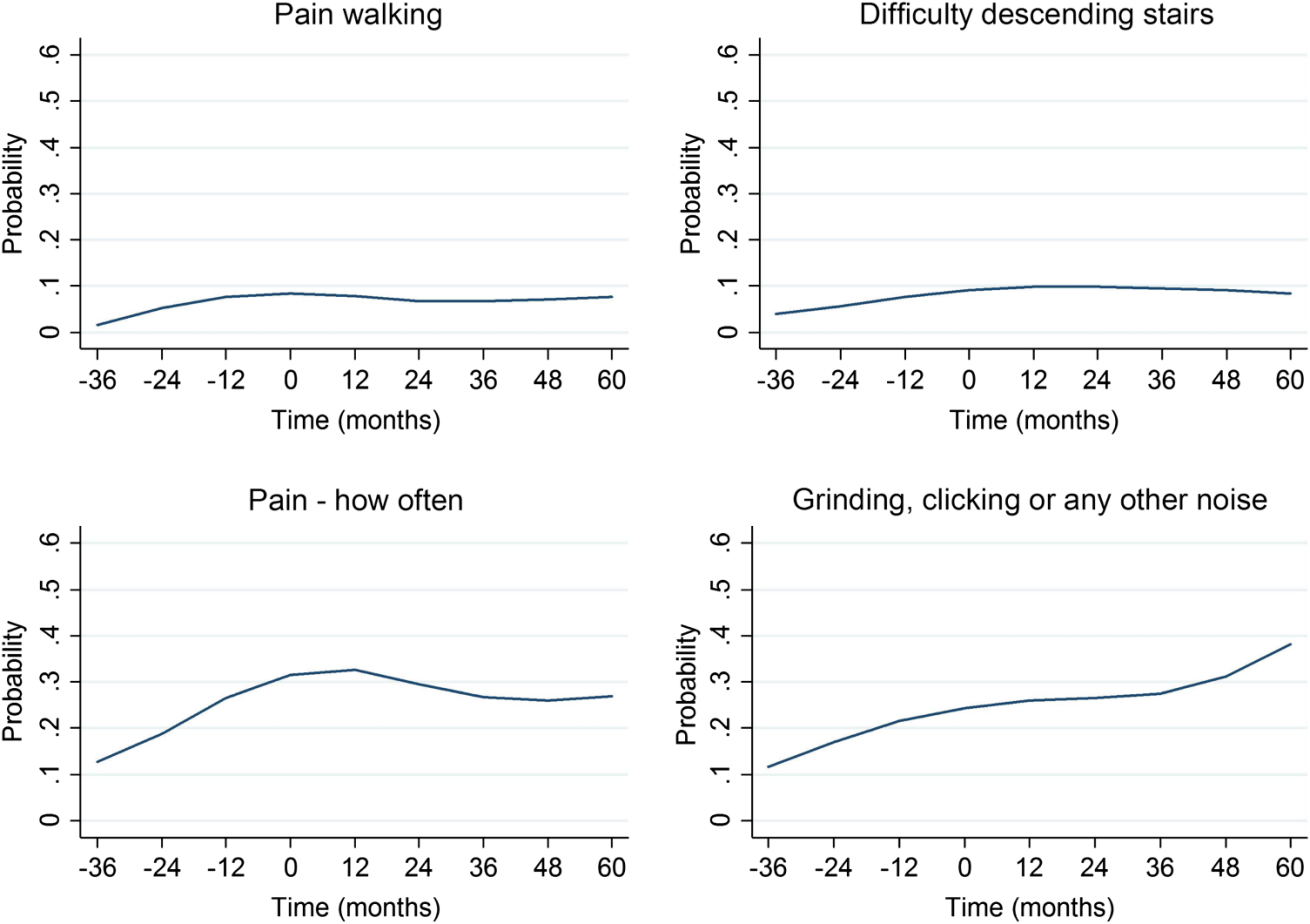
Example of trajectories for individual items: data from the Osteoarthritis Initiative, USA, 2004–2012 (from top left to bottom right: probability of scoring moderate, severe or extreme (pain walking and difficulty descending stairs), probability of experiencing pain weekly, daily or always and probability of feeling grinding, hearing clicking or any other type of noise sometimes, often or always. Time 0 represents the time of incident radiographic knee osteoarthritis).

The probability of having at least one symptom in each of the subscales generally stabilised or lessened following the initial prodromal phase with mostly non-significant associations of time after t0, for example, ORs for WOMAC Stiffness peak at t0 (OR at t0+1: 0.55 (95% CI 0.31 to 0.97)), suggesting that stiffness symptoms reduce in the year after incidence. Similar results were found across the individual items (data not shown). Two items to note were pain frequency (weekly or more; measured in KOOS), which increased greatly in the lead up to incident ROA and then decreased slightly, and audible joint sounds ( sometimes or more; measured in KOOS), which had a much higher overall probability, and after increasing prior to incident radiographic OA, stabilised then increased again at 5 years (figure 1). On removing knees which progressed to a higher KL grade up to 3 years after t0 (n=22, 13%), there was very little change to the trajectories (data not shown).

## Discussion

Following an increase in the risk of symptoms during the prodromal phase, this risk does not continue to increase in the period up to 5 years after the incidence of ROA. Instead, on most measures, it appears to stabilise or lessen, but not to prior levels. Reasons for the observed stabilisation or reduction of symptoms after incident ROA include the possible effect of treatment or, as alluded to by Hutton,^8^ a process of adaptation at the level of the joint and/or individual. An important caveat is that this may apply only in the absence of further disease progression since relatively few knees in this analysis progressed to KL grade ≥3 during the period of observation after incident ROA.

While undergoing the transition to incident ROA, a high proportion of knees were not at any time reported as having ‘moderate’ or worse symptoms: 50% of knees did not have ‘moderate’, ‘severe’ or ‘extreme’ symptoms on any individual items in the WOMAC Stiffness subscale; 36% on WOMAC Physical Function subscale; 43% on WOMAC Pain; 20% on KOOS Pain and 25% on KOOS Other Symptoms. Only 3–10% did not have even ‘mild’ symptoms on at least one item. The transition to incident ROA is therefore not entirely ‘silent’, but symptom changes may be subtle and may not trigger help-seeking. Rather than immediate clinical application, our findings serve to advance our understanding of the temporal relationship between symptom change and disease progression, raising the possibility of episodic symptom worsening in response to an underlying transition in disease state.

We have studied prodromal^2^ and postdromal symptom trajectories anchored around the transition to incident tibiofemoral ROA (KL grade ≥2). Extending this approach to earlier, preradiographic index events and states based on MRI and identifying preventable proximal triggers (eg, recent injury) is warranted.
